# Microbiome research outlook: past, present, and future

**DOI:** 10.1093/procel/pwad031

**Published:** 2023-05-23

**Authors:** Yunyun Gao, Danyi Li, Yong-Xin Liu

**Affiliations:** Shenzhen Branch, Guangdong Laboratory of Lingnan Modern Agriculture, Genome Analysis Laboratory of the Ministry of Agriculture and Rural Affairs, Agricultural Genomics Institute at Shenzhen, Chinese Academy of Agricultural Sciences, Shenzhen 518120, China; R-Institute Co. Ltd., Beijing 100011, China; Shenzhen Branch, Guangdong Laboratory of Lingnan Modern Agriculture, Genome Analysis Laboratory of the Ministry of Agriculture and Rural Affairs, Agricultural Genomics Institute at Shenzhen, Chinese Academy of Agricultural Sciences, Shenzhen 518120, China

With its critical role in human health and disease, the microbiome has transformed modern biology. Over the past few years, microbiome research has evolved rapidly, with microbiologists gradually shifting their focus from cataloging microorganisms in the human microbiome to understanding their functional roles and how they interact with the host. Here, we present the global trends in microbiome research and summarize the past and current work on microbiome published in *Protein* & *Cell*. In closing, we highlight some of the major advancements in microbiome research, including technical, practical, and conceptual advancements, that aim to enhance disease diagnosis, medicine development, and personalized interventions.

## Global trends and advancements in microbiome research: from composition to functionality

**Figure 1.**
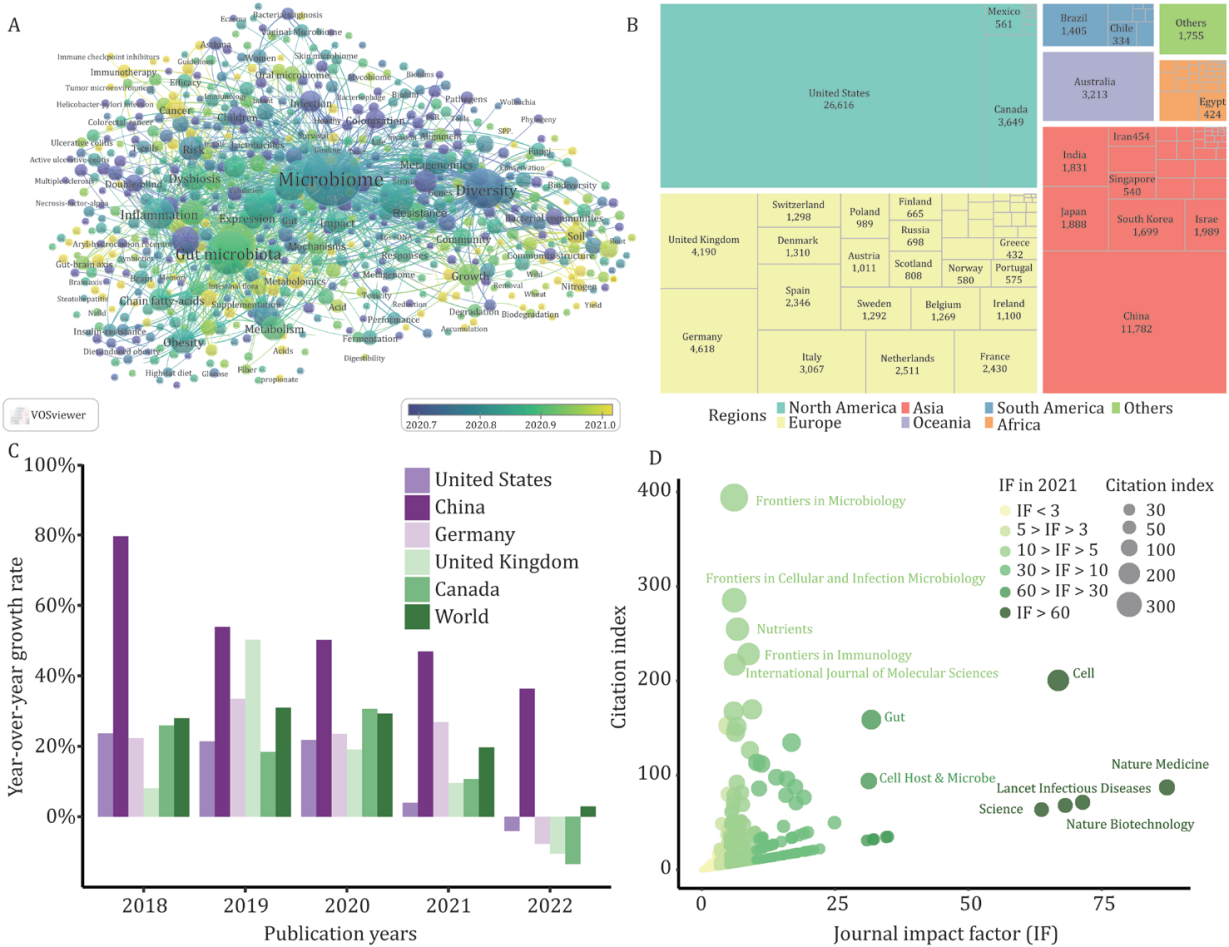
**The trends and growth of microbiome research over the past years.** Data were extracted from Web of Science Core Collection database on March 29th, 2023. (A) Keywords cluster map illustrating the hot spots in microbiome research, with 638 terms occurred at least 100 times since 1985. (B) Tree map indicating the geographical distribution of publications related to microbiome since 1985. (C) Bar graph displaying the year-over-year growth rate of the five countries with the most microbiome-related publications over the past 5 years. Year-over-year growth rate is calculated as follows: (the amount of the year’s publications—the amount of the last-year’s publications)/the amount of the last-year’s publications × 100%; (D) Bubble plot showing the citation indexes of journals citing publications from the first two special issues of *Protein* & *Cell*. Citation index is calculated as follows: the number of citations that a journal has made to the publications × the journal impact factor (IF) in 2021. (A) is drawn by VOSviewer.

Microbiome research has experienced major technical, practical, and conceptual progress in recent years (Lynch et al., 2019; Liu et al., 2022). During the early days, the initial focus was to describe the composition and diversity of microbiomes and to identify correlations between microbes and host phenotypes. However, current research has shifted toward refined studies dedicated to gaining functional insights into the microbiome, deciphering its mechanisms of action, and studying its co-evolution with the host (Li et al., 2022). These ongoing efforts will advance our understanding of how the microbiome influences various aspects of the host (Fig. 1A).

According to a search of the Web of Science Core Collection (data extracted on March 29th, 2023), most global research publications on the microbiome originated from North America, Asia, and Europe (Fig. 1B), and over two-thirds of the publications were released in the past 5 years. Notably, scientists from China have made an enormous contribution to the field since 2018, with a sustained increase in the year-over-year growth rate of publications (Figs. 1C and S1).

*Protein* & *Cell* offers a platform for publishing high-quality research and reviews on the microbiome. In the journal’s first two microbiome-themed special issues, several papers have received widespread acclaim worldwide (Fig. S2A), top of which are two reviews on the roles of the gut and oral microbiomes in disease diagnostics and therapy (Gao et al., 2018; Wang and Zhao, 2018), as well as a pipeline for acquiring microbiome data (Liu et al., 2020). These special issues have garnered more than 1,500 citations since 2018, with an average citation rate over three times that of other publications in this journal of the same period (Fig. S2B), and have featured contributions to publications in esteemed journals such as *Science*, *Cell*, and *Nature Medicine* (Figs. 1D and S2A).

## Unraveling the mysteries of the microbiome: impact on human health and disease

**Figure 2.**
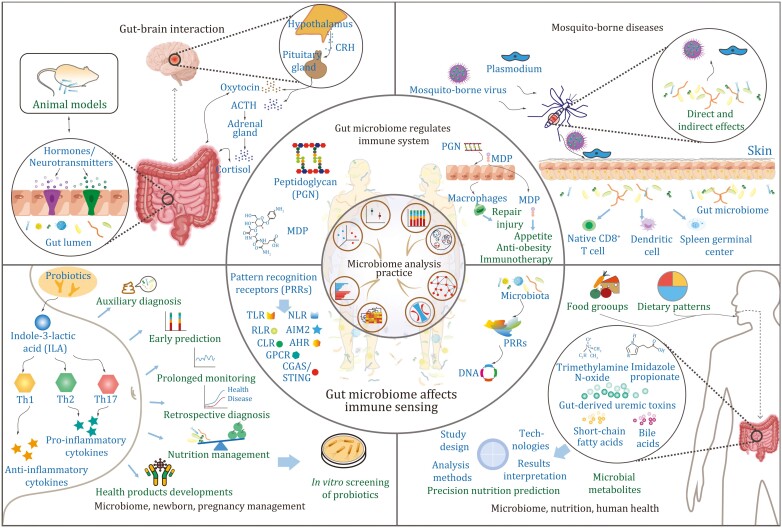
**The framework of the current microbiome special issue.** This framework covers seven important aspects of microbiome research, including best practices for analyzing microbiomes [inner circle, Wen et al. (2023)], the regulation of gut microbiomes on the human immune system [upper outer circle, Yin et al. (2023)], how gut microbiomes affect human immune sensing (lower outer circle, Wan et al. (2023)), the role of microbiomes in gut–brain interaction [upper left, Wang et al. (2023), Zheng et al. (2023)], how microbiomes can interfere with the spread of mosquito-borne diseases in both mosquitoes and humans (upper right, Shi et al. (2023)), the application of microbiomes in maternal and newborn health [lower left, Gao and Wang (2023)], and the study of nutri-microbiome epidemiology [lower right, Gou et al. (2023)]. The gut and brain of the figure are drawn with Figdraw.

In the current special issue of *Protein* & *Cell*, scientists are seeking to unravel the mysteries of microbiome on human health and disease (Fig. 2). Prof. Hongwei Liu characterizes the chemical structures of gut bacterial cell wall-derived molecules and proposes the role of microbiome-derived components in the training of the human immune system (Yin et al., 2023). Prof. Shu Zhu summarizes how components of gut microbiome and microbiota-derived metabolites are sensed by the host immune system and their involvement in the maintenance of human gut homeostasis (Wan et al., 2023). Gut microbiota plays an important role in gut–brain interactions, and two reviews discuss the underlying mechanisms, with Prof. Xingyin Liu focusing on the current research in neurodevelopmental disorders (Wang et al., 2023) and Prof. Liping Duan emphasizing on the study of irritable bowel syndrome (Zheng et al., 2023). Prof. Gong Cheng reviews the mechanisms of both the mosquito and host microbiomes against mosquito-borne diseases (Shi et al., 2023). Prof. Jinfeng Wang reconceptualizes the concept of maternal and infant health from a microbiome perspective (Gao and Wang, 2023). Prof. Jusheng Zheng provides extensive insights into the interplay between nutrition and microbiome for human health (Gou et al., 2023).

Meanwhile, to effectively manage the expanding volume of microbiome data and complex statistical software, a systematic approach is crucial for sorting and interpreting the results. Prof. Yong-Xin Liu conducts a comparative analysis and organization of commonly used R packages for analyzing microbiome data, culminating in a selection of tools that are both capable and efficient, also readily accessible to microbiome researchers (Wen et al., 2023).

## Exploring the frontiers of microbiome research: unlocking the advanced tools to revolutionize human health

**Figure 3.**
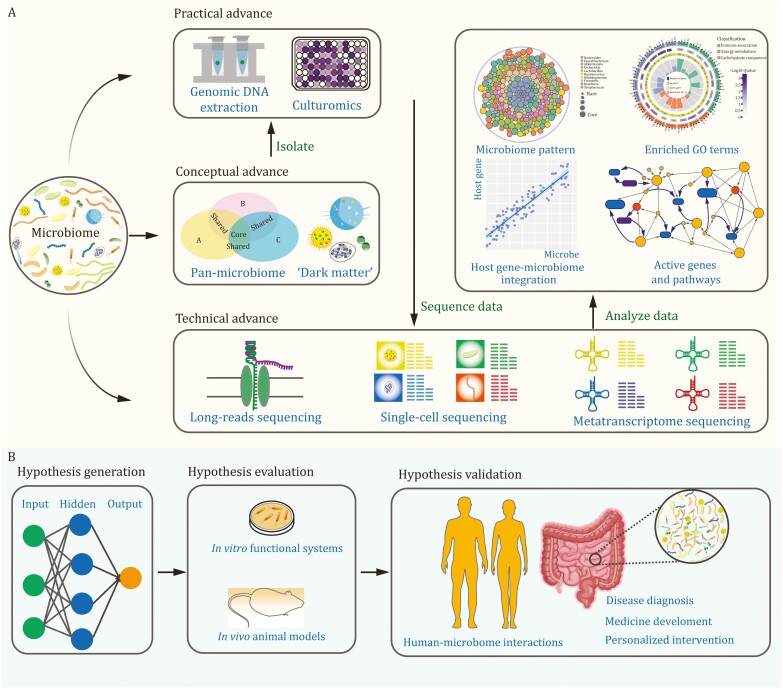
**Future direction of microbiome research.** (A) The flow chart presents an overview of the practical, conceptual, and technical advances in microbiome for studying its function and mechanisms. (B) The research data of microbiome is essential for hypothesis generation, evaluation, and validation.

Soon, we believe that there will be major advancements in microbiome research, owing to practical, conceptual, and technical improvements. The practical improvements in high-molecular-weight DNA extraction (Maghini et al., 2021) and high-throughput culturomics techniques (Zhang et al., 2021) will revolutionize the workflow of microbiome research, facilitating extensive study of the functions and mechanisms of the microbiome. With the ever-increasing number of studies, a focus on understanding the microbiome patterns in health and disease will continue to be a crucial area of investigation (Wilmanski et al., 2021). The pan-microbiome concept will be a promising strategy for identifying the core microbiome and microbiome patterns, which will be helpful to explain and understand the observed heterogeneity among individual hosts. And greater attention should be put on studying the “dark matter” of the microbiota such as fungi, viruses, archaea, and protozoa in the coming years, as these microorganisms have been largely overlooked in the past but may have as critical roles as that of bacteria. Furthermore, the introduction of cutting-edge sequencing technologies, such as long-read sequencing, single-cell sequencing, and metatranscriptomic sequencing, will allow higher resolution at the level of species or strain (Fig. 3A). And the development of cloud platforms (Chen et al., 2021, 2022) and R packages (Wen et al., 2022) will greatly facilitate data exploration in microbiome research in terms of analysis.

Based on these developments, it is conceivable that more hypotheses regarding the relationships between microbiome and host will be generated, and *in vitro* and *in vivo* experiments should be conducted before validating them in humans (Fig. 3B). Ultimately, we may use the microbiome as a versatile tool for disease diagnosis, medicine development, and personalized interventions. Upon unlocking this toolkit, we are on the cusp of a revolution in microbiome research that will transform the field and improve human health in unprecedented ways.

## Supplementary Material

pwad031_suppl_Supplementary_Figures

## Data Availability

No new sequencing data generated by this project.

## Code availability

All data and draw code are available in GitHub github.com/YunyunGao374/ProteinCell2023MicrobiomeReview.
