# Supplementary material for: Microbiome research outlook: past, present, and future
Source: Protein Cell. 2023 May 23;14(10):709–12. doi: 10.1093/procel/pwad031 (PMC10599639; doi:10.1093/procel/pwad031)
Supplement: pwad031_suppl_Supplementary_Figures [file pwad031_suppl_supplementary_figures.pdf]

# Supplementary Figures

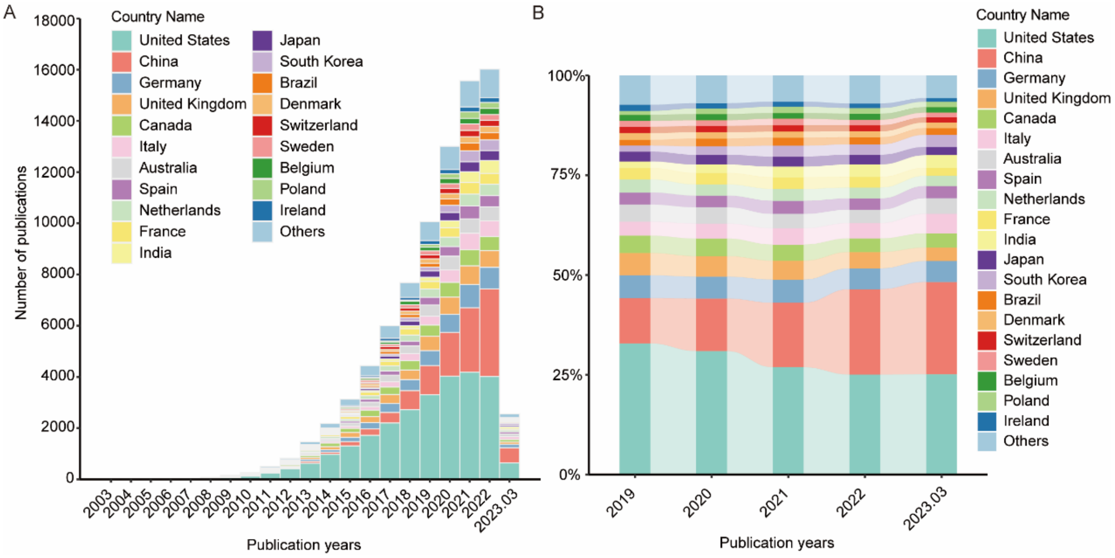


**Figure S1. Microbiome publications in the top 20 countries.**

Data were extracted from Web of Science Core Collection database on March 29, 2023.

(A) The number of publications in different countries every year, starting from 2003. (B) The percentage of publications from different countries in the past five years.

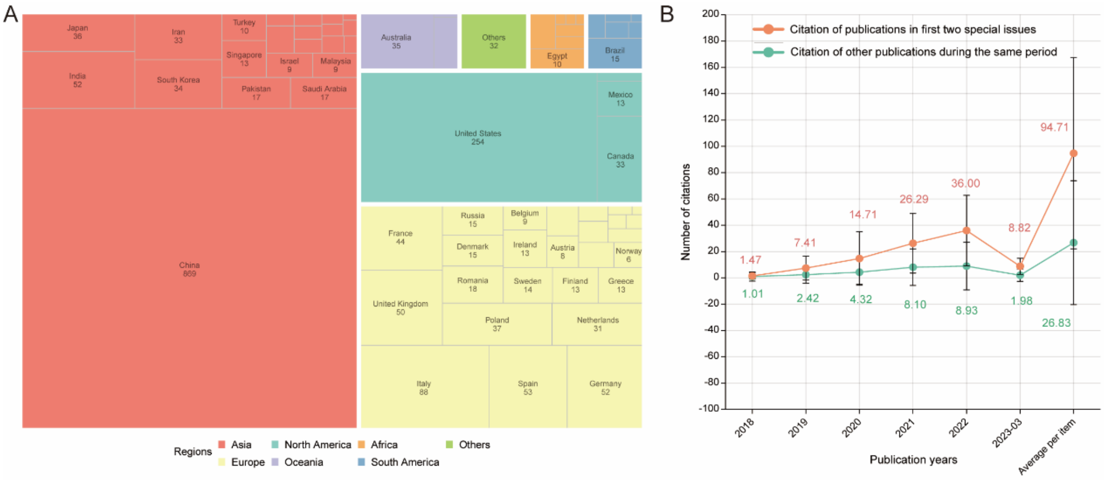


**Figure S2. Citations of literature published in *Protein & Cell* microbiome special issues.**

Searched by Web of Science Core Collection database until March 29, 2023.

(A) The global distribution of citations of the research published in the first two microbiome-themed special issues of *Protein & Cell*. (B) The average citation numbers of literature published in first two special issues and other publications in this journal during the same period after publication.
